# Supplementary material for: Structural mechanisms for the recruitment of factor H by Streptococcus pyogenes
Source: bioRxiv. 2025 Aug 5:2025.08.05.668778. Preprint. [Version 1] doi: 10.1101/2025.08.05.668778 (PMC12340840; doi:10.1101/2025.08.05.668778)
Supplement: Supplement 1 [file media-1.pdf]

Structural mechanisms for the recruitment of factor H by *Streptococcus pyogenes*

Amit Kumar, Kuei-Chen Wang, and Partho Ghosh

Supplemental Figures S1-S13

Supplemental Tables S1-S3

Supplemental Figure S1

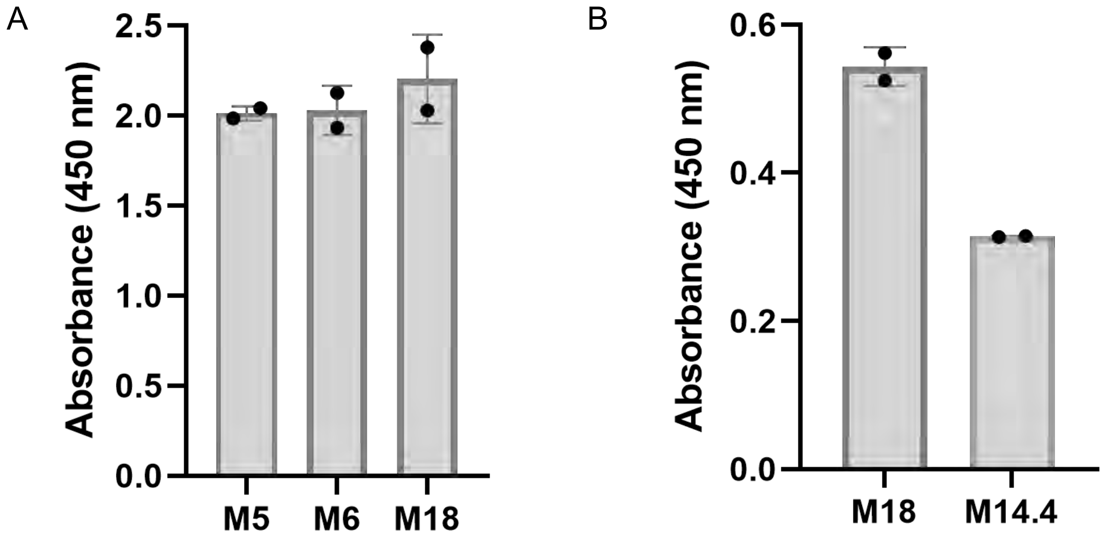

Figure S1. FH binding to M proteins.
A. Binding of soluble, intact FH to immobilized intact His_6-M5, His_6-M6, and His_6-M18 proteins, as evaluated by ELISA. Bound FH was detected with an anti-FH monoclonal antibody. Data from two biological replicates are presented with means and standard deviations.
B. Same as panel A, but for His_6-M18 and His_6-M14.4 proteins.

Supplemental Figure S2

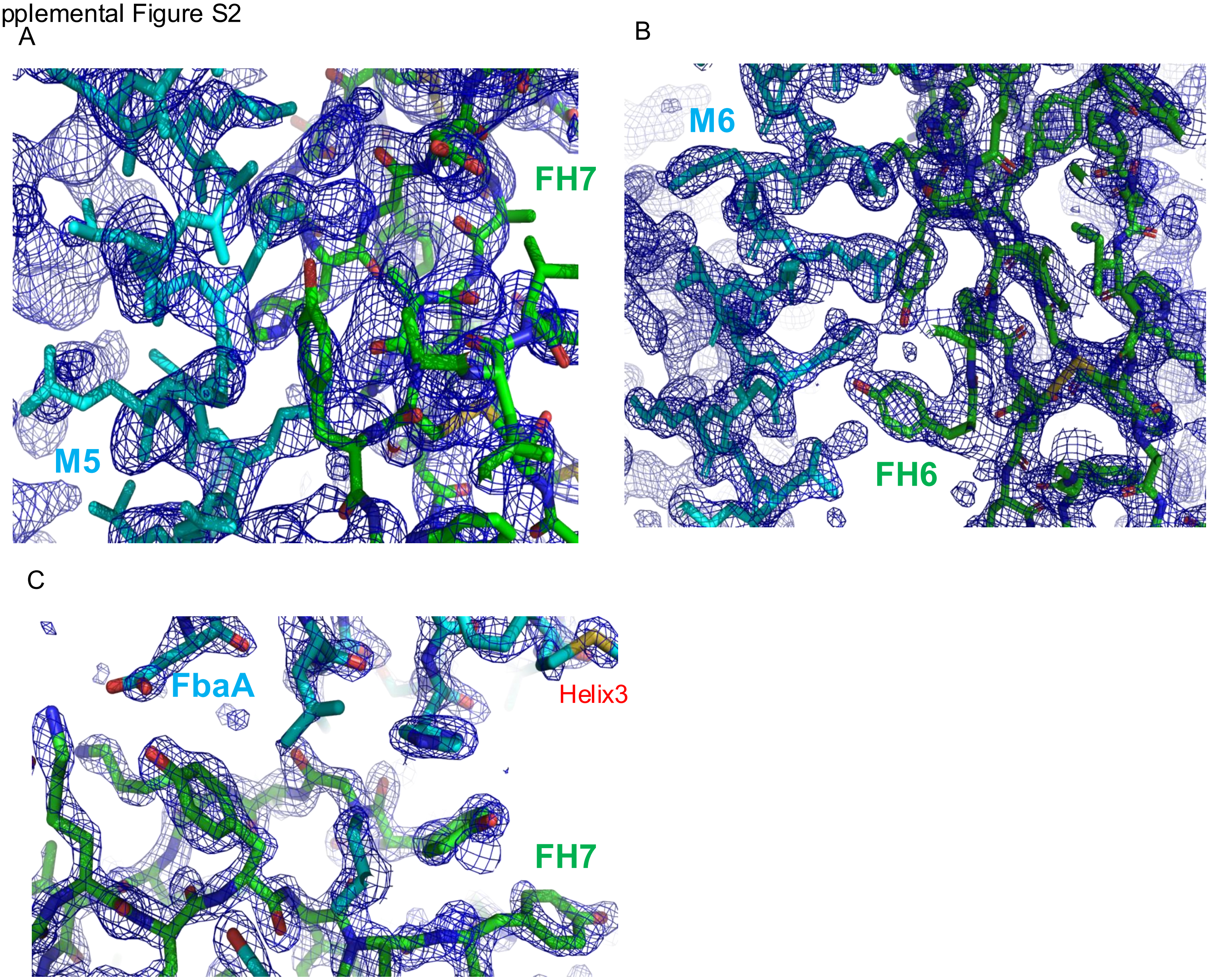

Figures S2. Electron Density. Composite omit 2mFo-DFc electron density map contoured at 2σ for the (**A**) M5/FH(6-7), (**B**) M6/FH(6-7), and (**C**) FbaA/FH(6-7) complexes. M5 protein, M6 protein, and FbaA are in cyan, and FH in green.

Supplemental Figure S3

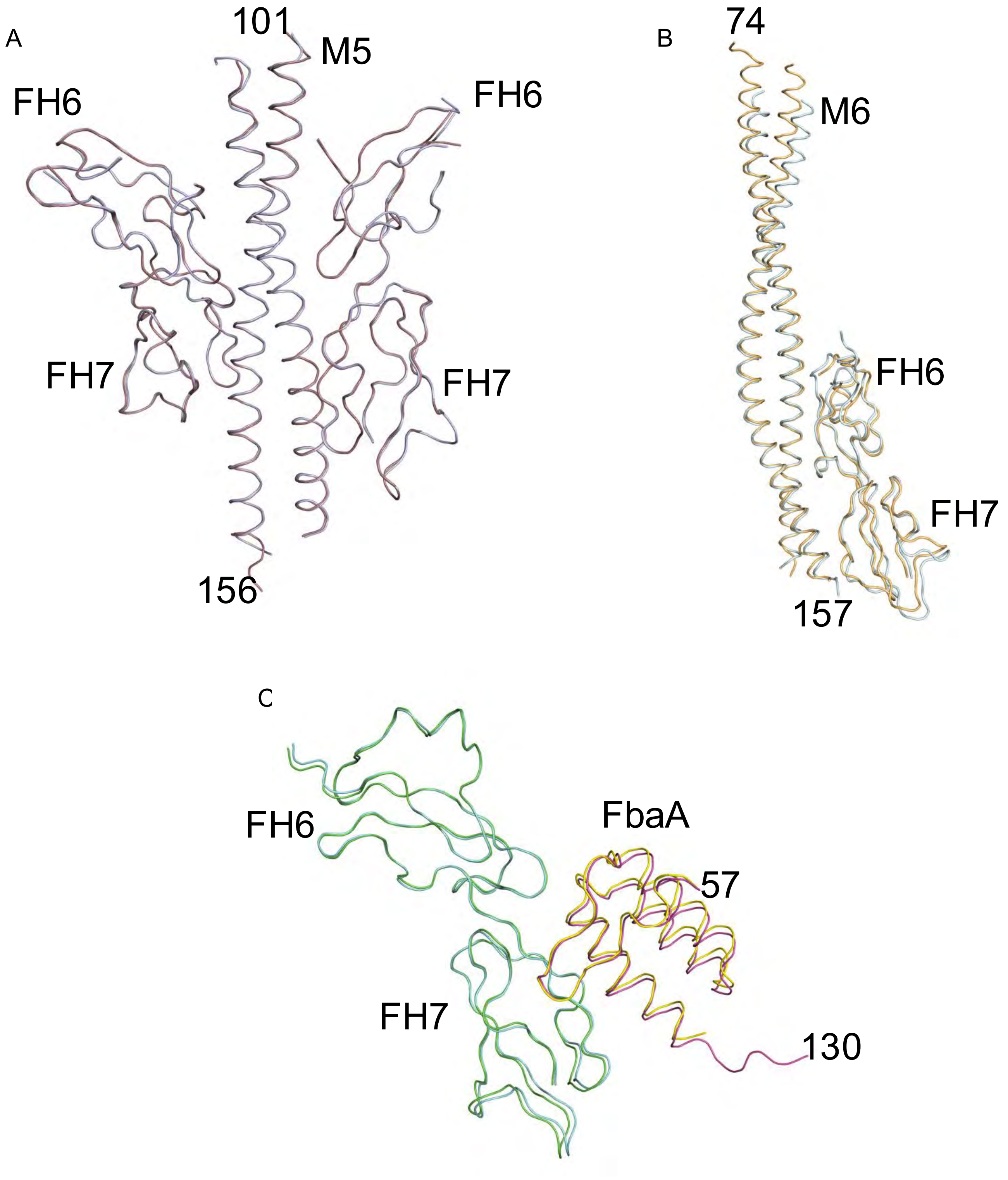

Figure S3. M protein-FH complexes in the asymmetric unit.
Superposition of the two (**A**) M5/FH(6-7), (**B**) M6/FH(6-7), and (**C**) FbaA/FH(6-7) complexes in the asymmetric unit of each respective crystal.

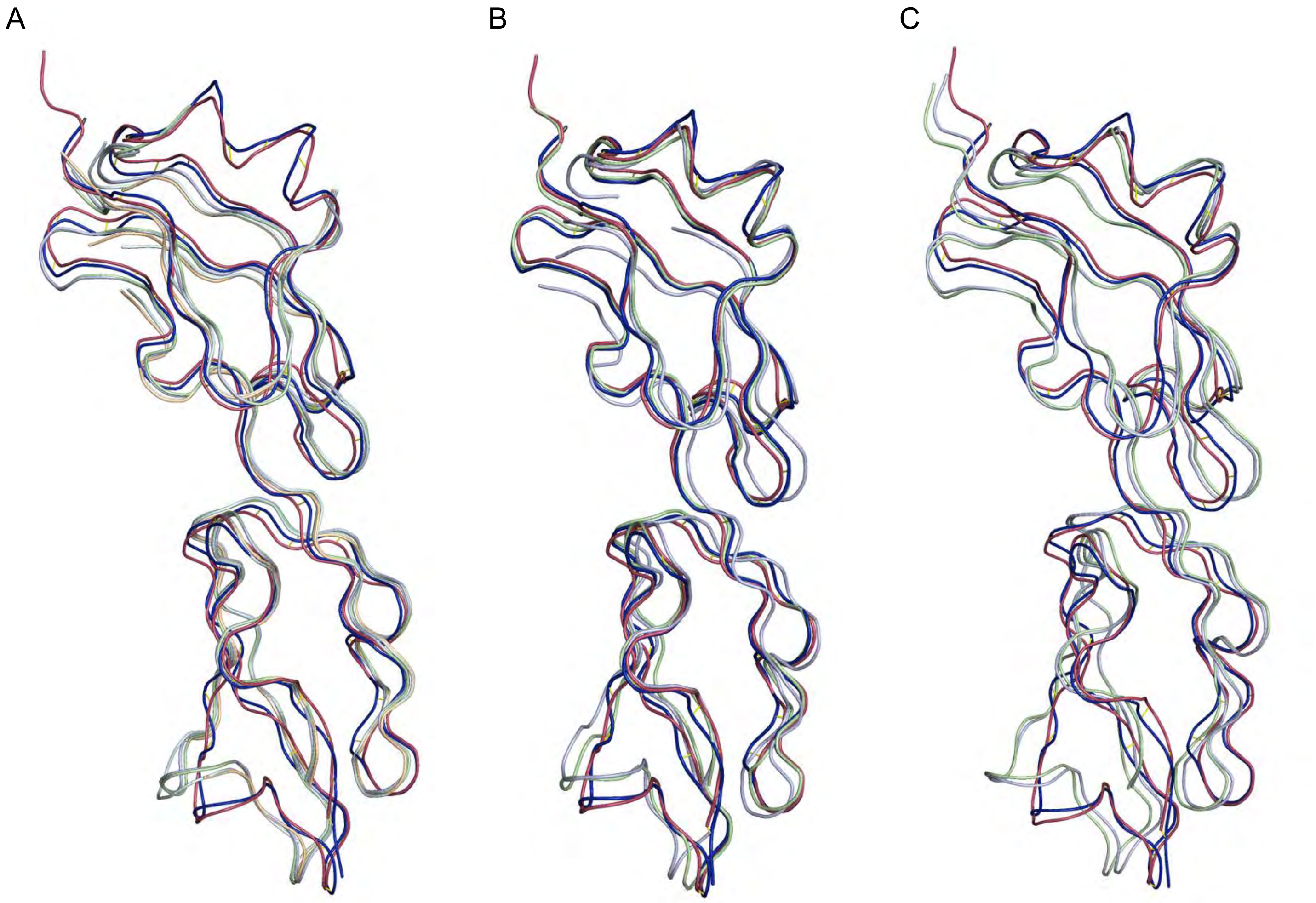

Figure S4. Stable conformation of FH(6-7). Superposition of FH(6-7) when bound to *N. meningitidis* fHbp (red, PDB:2w80), *B. burgdorferi* CspZ (blue, PDB: 7ZJM), and (**A**) M5 protein (green, cyan, light blue and wheat), (**B**) M6 protein (green and light blue), and (**C**) FbaA (green and light blue).

Supplemental Figure S5

A

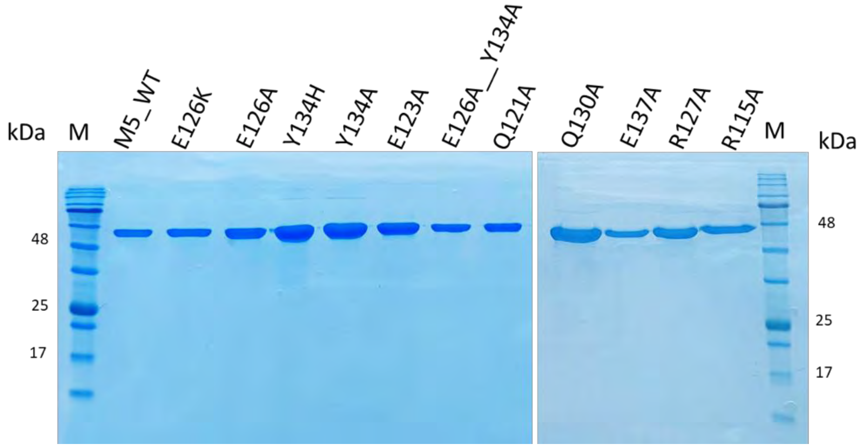

B

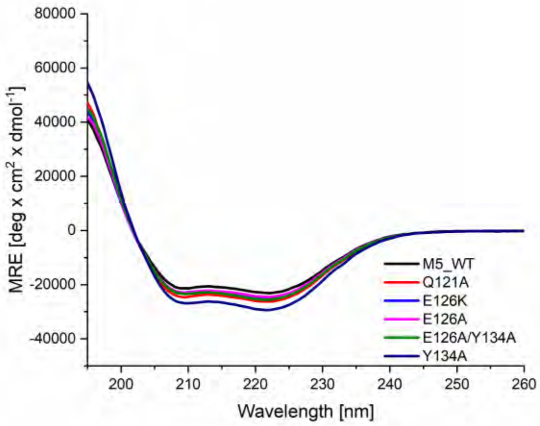

C

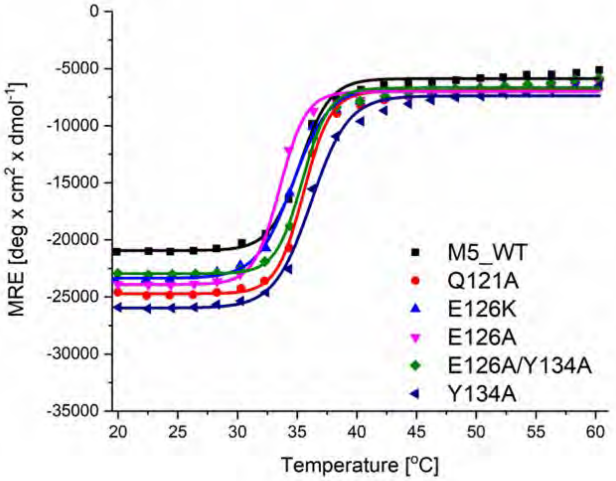

D

Name	Tm (oC)
M5_WT	35.85 ± 0.91
Q121A	34.66 ± 1.22
E126K	34.86 ± 0.31
E126A	34.79 ± 1.76
E126A/Y134A	35.74 ± 0.45
Y134A	36.17 ± 0

E

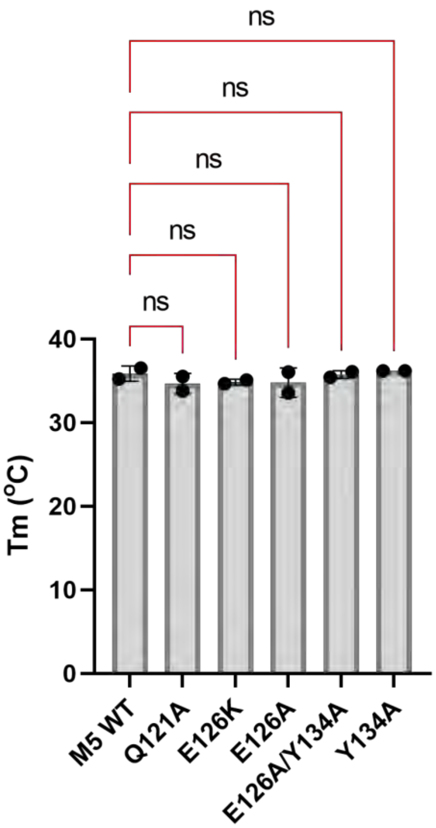

Figure S5. Structure and stability of wild-type and mutant His$_6$-M5 proteins.
A. SDS-PAGE of purified intact wild-type and mutant His$_6$-M5 proteins.
B. CD spectra of intact wild-type and mutant M5 proteins at 25 °C. The data are an average of two independent measurements.
C. Thermal melting curves of intact wild-type and mutant M5 proteins, as monitored by the CD signal at 222 nm. The data are an average of two independent measurements.
D. Tabulation of Tm's determined from panel C.
E. Statistical analysis of Tm's, determined by one-way ANOVA. P-value for ns > 0.123.

Supplemental Figure S6

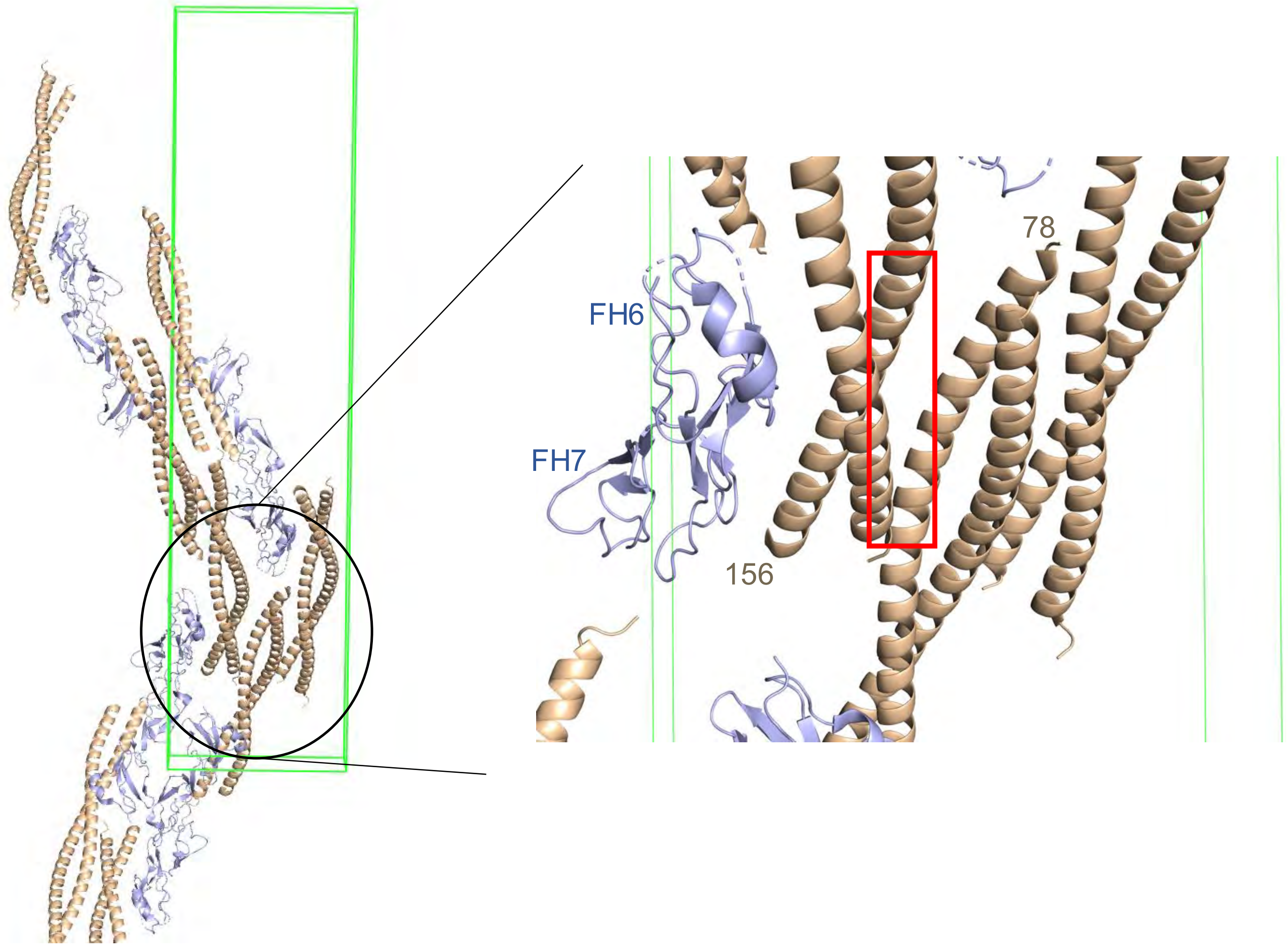

Figure S6. M6/FH(6-7) crystal packing. The green box indicates the unit cell, with M6 protein in wheat and FH(6-7) in light blue. The red box indicates an FH-binding site in M6 protein that is unoccupied and instead forms a contact with a crystallographically related M6 protein dimer.

Supplemental Figure S7

A

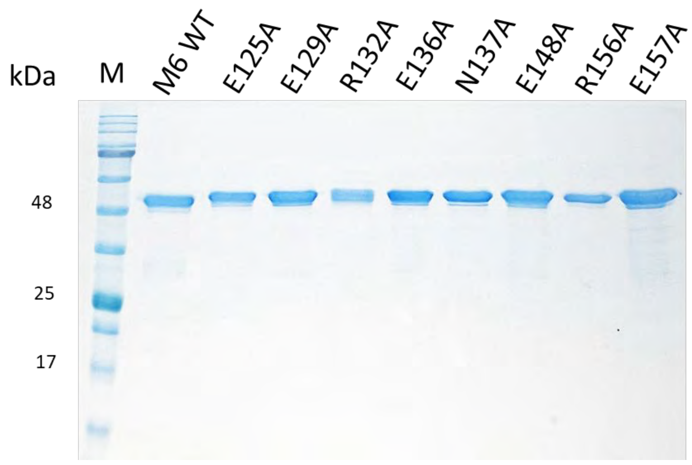

B

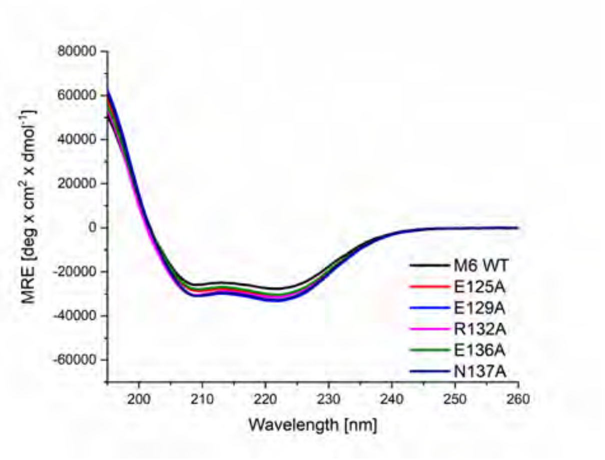

C

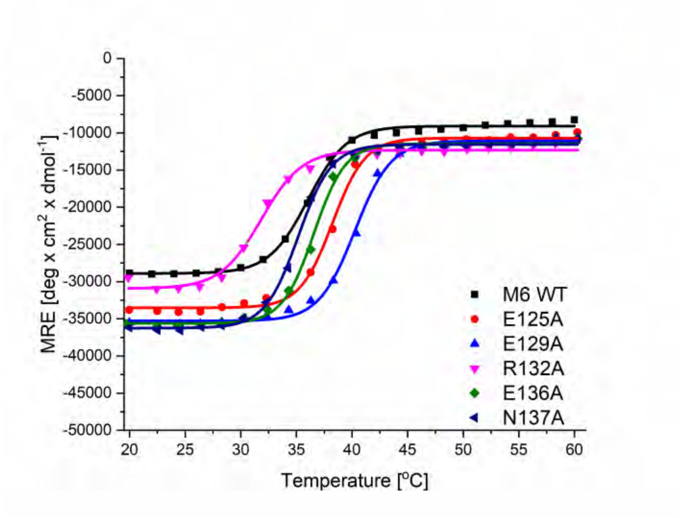

D

**Name**	**Tm (°C)**
**M6 WT**	36.12 ± 0.11
**E125A**	38.74 ± 0.79
**E129A**	39.78 ± 0.65
**R132A**	32.03 ± 0.28
**E136A**	37.43 ± 1.23
**N137A**	34.98 ± 0.25

E

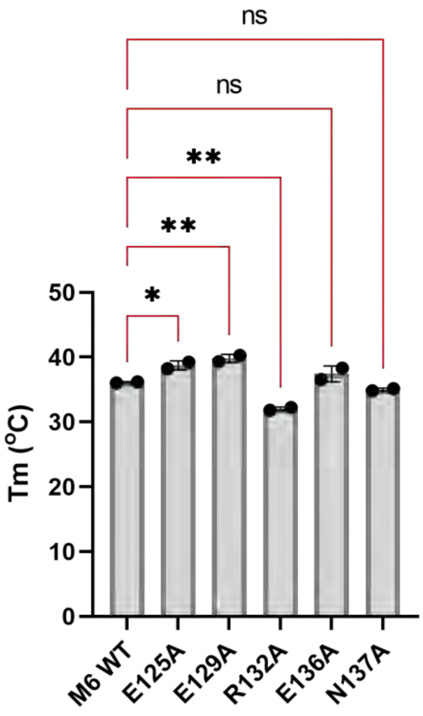

Figure S7. Structure and stability of WT and mutant His_6-M6 proteins.
A. SDS-PAGE of purified intact wild-type and mutant His_6-M6 proteins.
B. CD spectra of intact wild-type and mutant M6 proteins at 25 °C. The data are an average of two independent measurements.
C. Thermal melting curves of intact wild-type and mutant M6 proteins, as monitored by the CD signal at 222 nm. The data are an average of two independent measurements.
D. Tabulation of Tm's determined from panel C.
E. Statistical analysis of Tm's determined by one-way ANOVA. p-values are as follows: ns > 0.123, * ≤ 0.033, and ** ≤ 0.0021.

Supplemental Figure S8

A

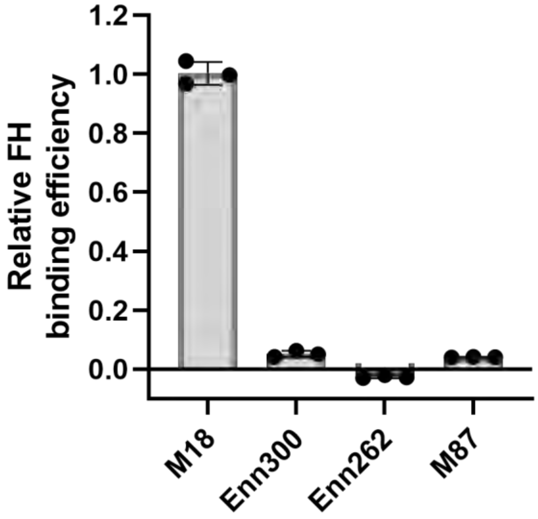

B

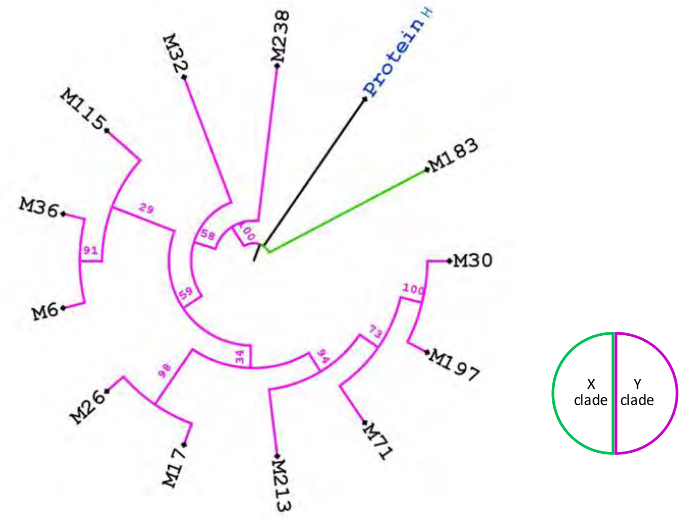

Figure S8. Threshold for M6 FH-binding pattern and phylogenetics.
A. Binding of soluble intact FH to immobilized intact His_6-M18, His_6-M87, His_6-Enn300, and His_6-Enn262 proteins, as evaluated by ELISA. Bound FH was detected with an anti-FH monoclonal antibody. Values were normalized by FH binding to His_6-M18 protein. Data from three biological replicates are presented with means and standard deviations.
B. Phylogenetic analysis of M proteins with M6 FH-binding pattern. Protein H was used as an outgroup.

Supplemental Figure S9

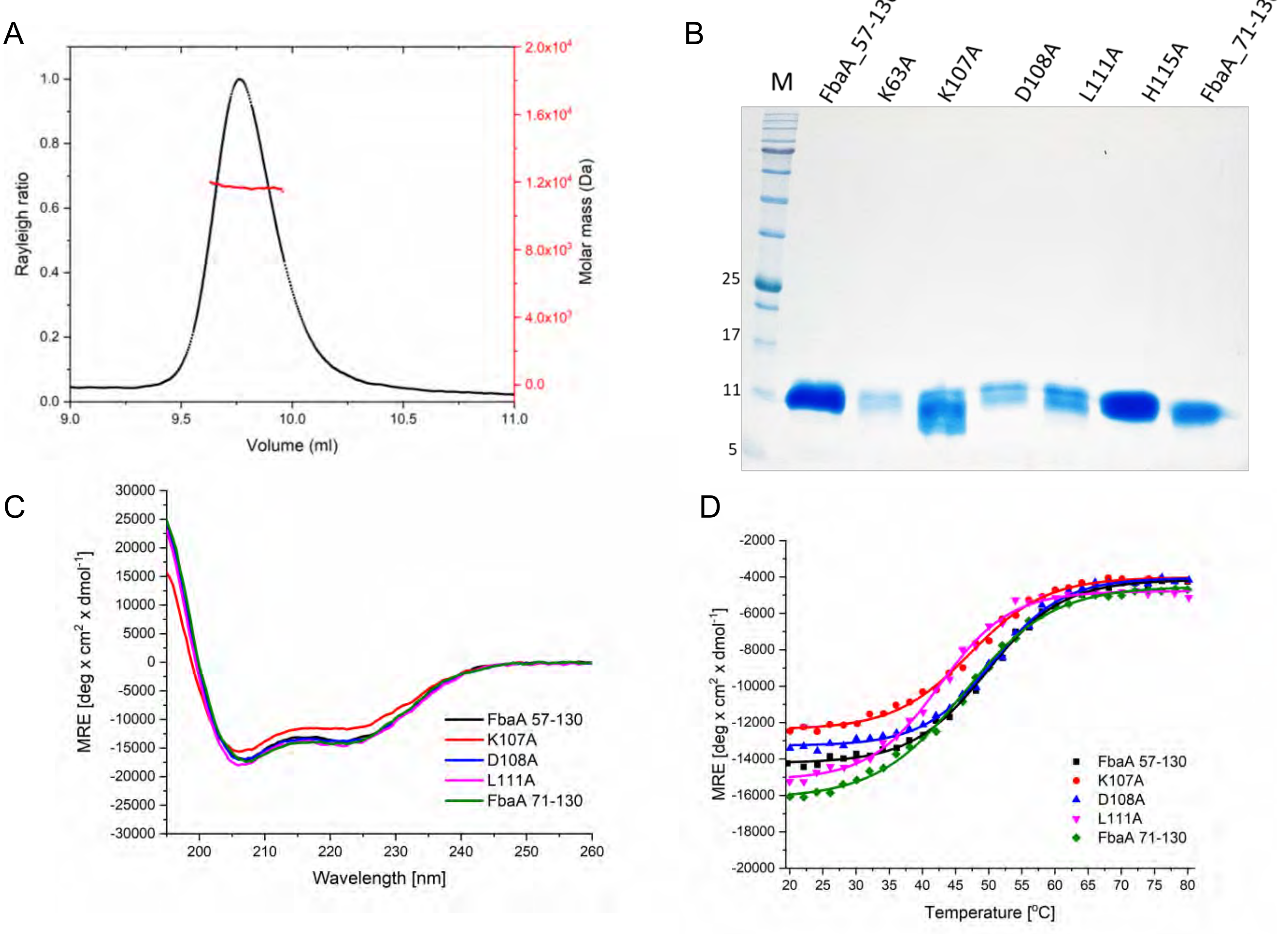

E

**Name**	**Tm (°C)**
**FbaA 57-130**	51.36 ± 2.31
**K107A**	48.92 ± 2.43
**D108A**	52.75 ± 3.00
**L111A**	44.00 ± 1.86
**FbaA 71-130**	48.00 ± 2.42

F

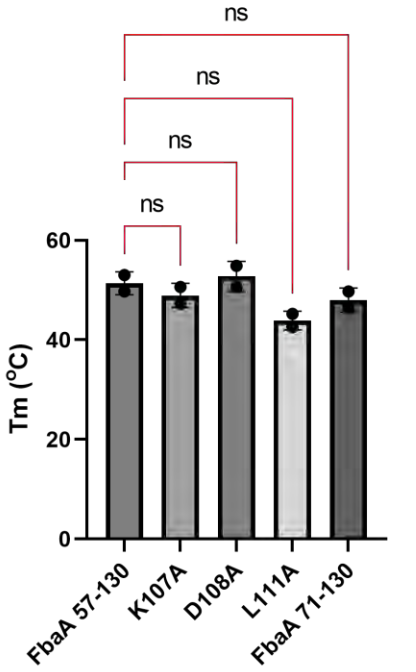

Figure S9. Wild-type and mutant FbaA (57-130).
A. Molecular mass of FbaA (57-130) determined by SEC-MALS.
B. SDS-PAGE of wild-type and mutant His_6-FbaA (57-130).
C. CD spectra of wild-type and mutant FbaA (57-130) at 25 °C. The data are an average of two independent measurements.
D. Thermal melting curves of intact wild-type and mutant FbaA (57-130), as monitored by the CD signal at 222 nm. The data are an average of two independent measurements.
E. Tabulation of Tm's determined from panel D.
F. Statistical analysis of Tm's, determined by ordinary one-way ANOVA. p-value for ns > 0.123.

Supplemental Figure S10

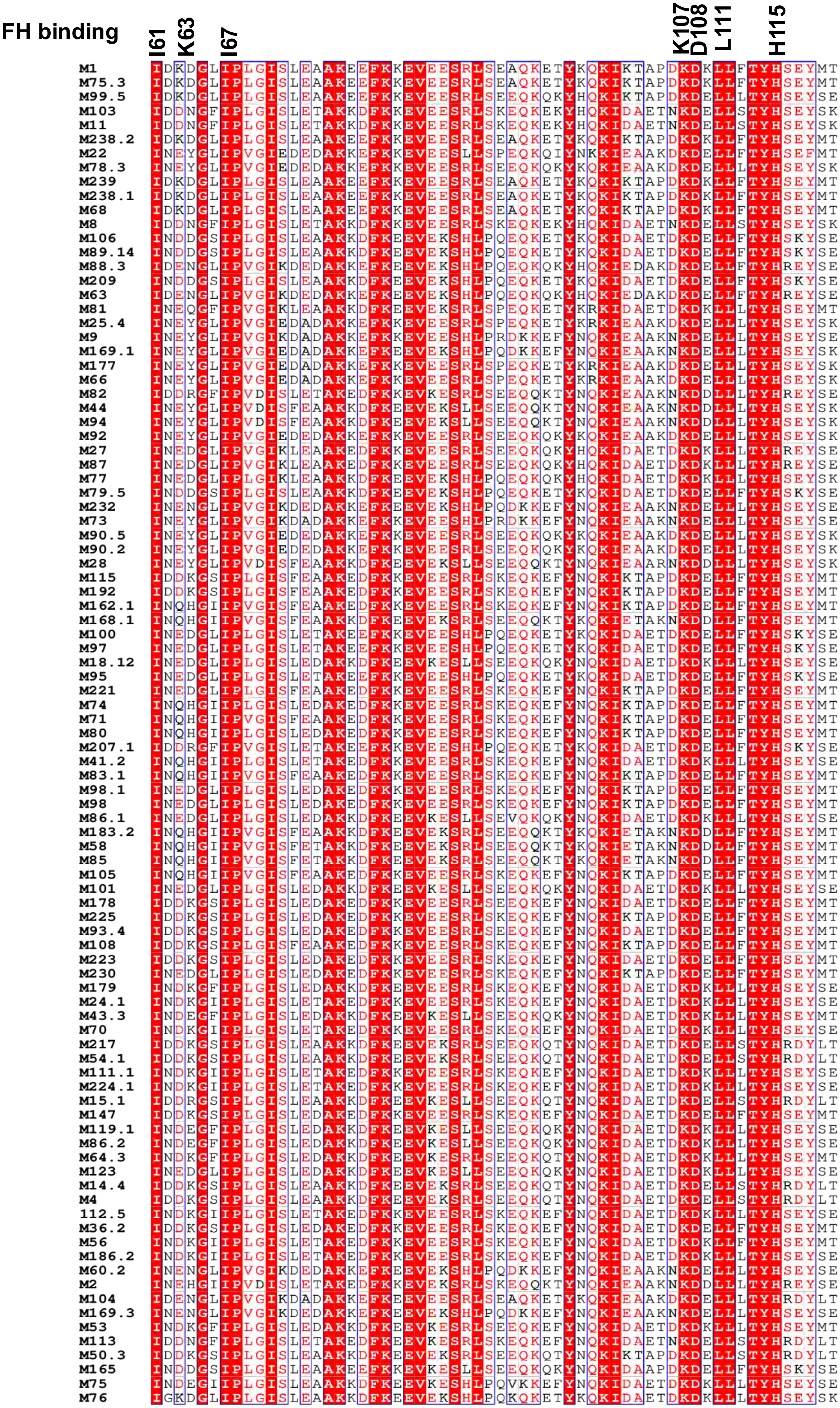

Figure S10. FH-binding sequence in FbaA.

Sequence alignment of the loop and three-helix bundle region from FbaA belonging to the M1 strain with FbaA from other M types. FH-interacting amino acids at the top.

Supplemental Figure S11

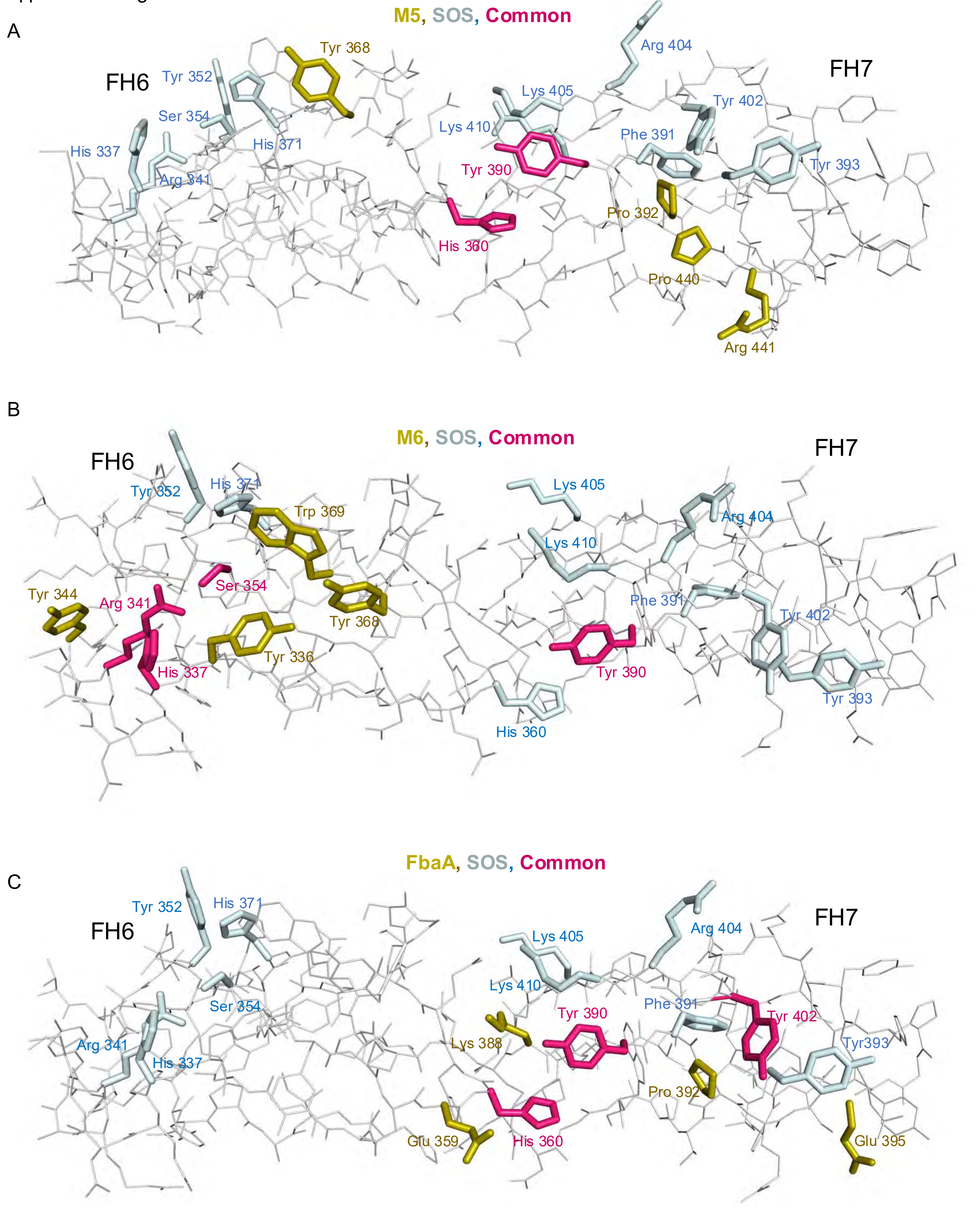

Figure S11. Common FH amino acids for SOS.
A. FH domains 6 and 7 with side chains that contact only M5 protein in gold, only SOS in pale blue, and those in common in red.
B. The same as panel A, but for M6 protein.
C. The same as panel A, but for FbaA.

Supplemental Figure S12

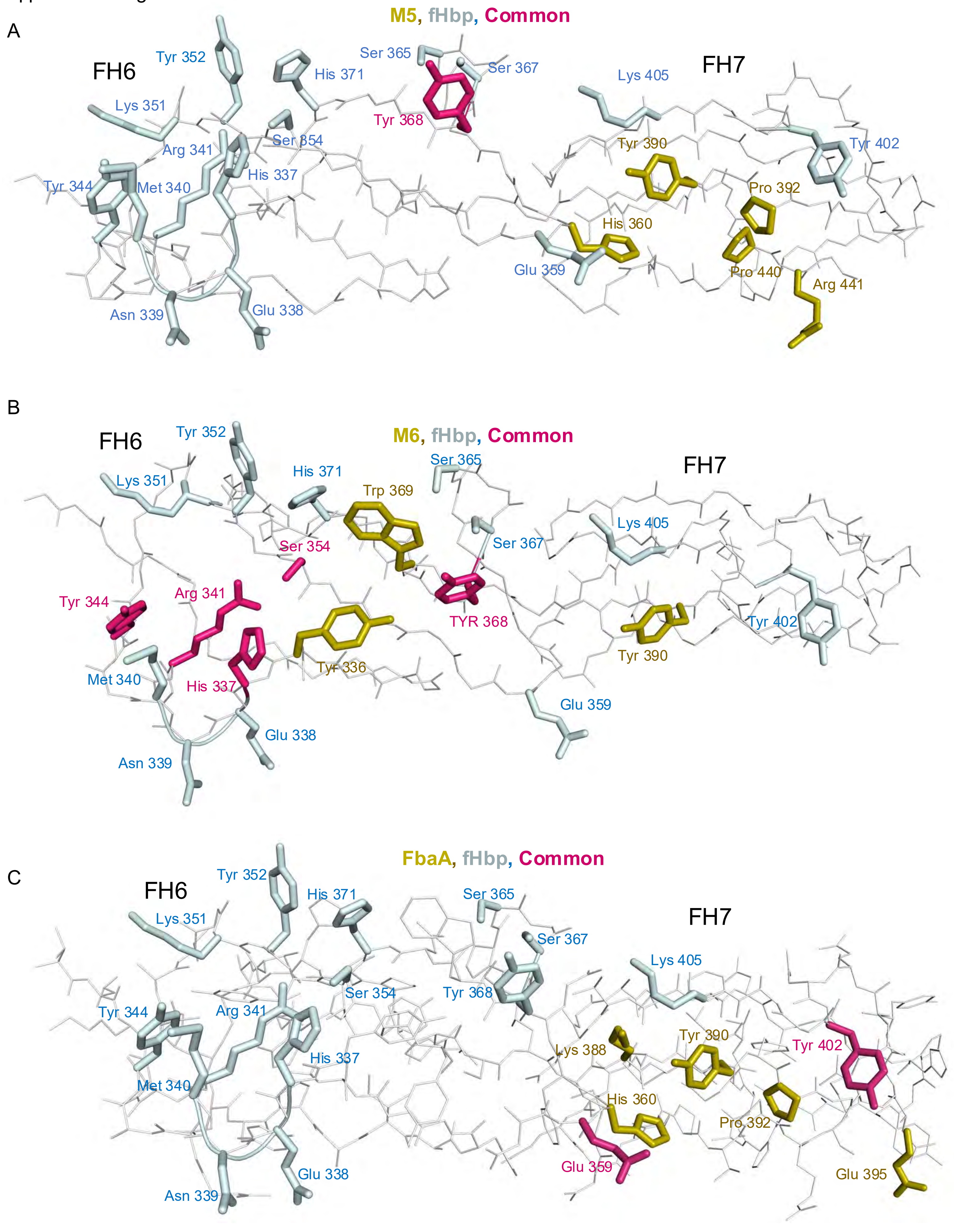

Figure S12. Common FH amino acids for fHbp.

A. FH domains 6 and 7 with side chains that contact only M5 protein in gold, only fHbp in pale blue, and those in common in red.

B. The same as panel A, but for M6 protein.

C. The same as panel A, but for FbaA.

Supplemental Figure S13

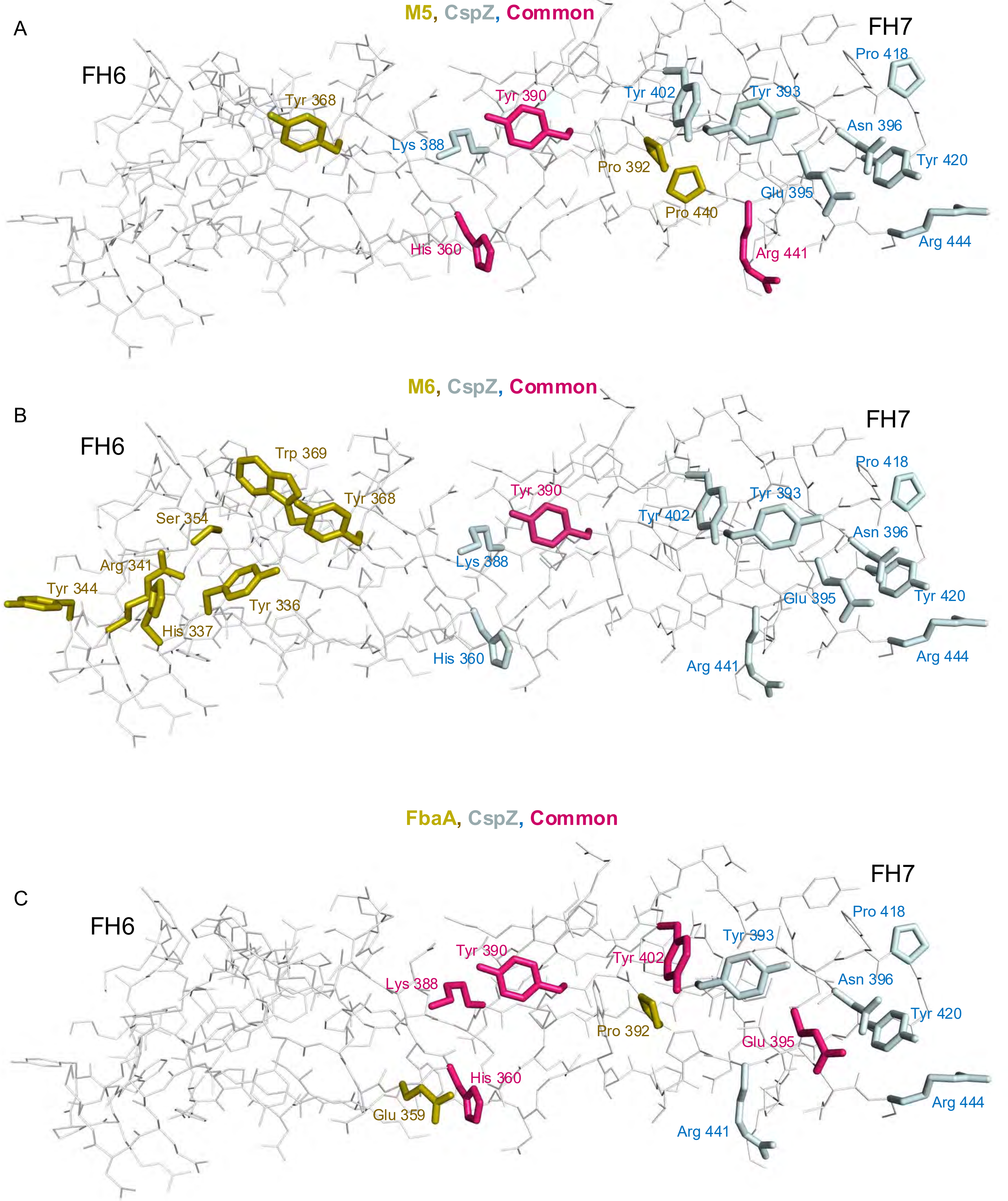

Figure S13. Common FH amino acids for CspZ.

A. FH domains 6 and 7 with side chains that contact only M5 protein in gold, only CspZ in pale blue, and those in common in red.

B. The same as panel A, but for M6 protein.

C. The same as panel A, but for FbaA.

Table S1. Crystallographic data collection and model refinement.

	M5-FH	M6-FH	FbaA-FH
**Data collection**			
Wavelength (Å)	1.0	1.0	1.54
Resolution range (Å)	50.00-2.25 (2.29-2.25)	44.90-1.90 (1.94-1.90)	36.19-1.82 (1.86-1.82)
Space group	$P\ 3_2$	$P\ 2\ 2_1\ 2_1$	$P\ 1\ 2_1\ 1$
**Cell dimensions**			
a, b, c (Å)	62.7, 62.7, 199.3	33.4, 69.5, 293.9	28.5, 79.0, 89.9
α, β, γ (°)	90.0, 90.0,120.0	90.0, 90.0, 90.0	90.0, 91.8, 90.0
Total reflections	266,494	711,806	69,389
Unique reflections	41,276	55,717	35,778
Redundancy	6.5 (5.6)	12.8 (12.7)	1.9 (1.9)
Completeness (%)	99.5 (98.7)	99.8 (99.9)	99.8 (99.6)
I/σ(I)	11.4 (0.5)	9.8 (0.5)	6.6 (1.6)
Rmeas	0.23 (1.859)	0.130 (5.955)	0.094 (0.809)
$CC_{1/2}$	0.948 (0.070)	0.999 (0.304)	0.996 (0.543)
Wilson B-factor ($Å^2$)	45.2	36.6	18.6
**Refinement**			
Resolution range (Å)	47.72 - 2.25	44.89 - 1.90	36.19 - 1.82
No. of reflections (work/test set)	41,213/4,081	55,577/5,535	35,742/3,568
Rwork/Rfree	0.253/0.287	0.260/0.302	0.226/0.275
No. of non-hydrogen atoms for Protein	4,544	4,075	3,016
No. of non-hydrogen atoms for Ligands	None	12 (ethylene glycol)	48 (MES)
No. of non-hydrogen atoms (water)	70	98	312
L-test for twinning*	< |L| > = 0.41, < L2 > = 0.23	< |L| > = 0.50, < L2 > = 0.33	< |L| > = 0.50, < L2 > = 0.33
**r.m.s. deviations**			
Bonds (Å)	0.0025	0.0050	0.0040
Angles (°)	0.47	0.69	0.79
**Ramachandran plot**			
Favored (%)/allowed (%)	95.15/4.85	99.08/0.92	98.96/1.04
Outliers (%)	0	0	0
Rotamer outliers (%)	1.81	0	0
Clashscore	6.87	3.87	4.33
Number of TLS groups	0	23	10
PDB code	9MMU	9MMX	9MLU

*Theoretical values of < |L| >, < L2 > for acentric reflections are 0.5, 0.333 respectively for untwined datasets, and 0.375, 0.2 for perfectly twinned datasets.

Table S2. M5 FH-binding pattern in M, Enn and Mrp proteins.
Red horizontal line indicates cut-off.

M	Position	Sequence	Score
**29**	**121**	**QRETLEKNNDDLTYKNDDL**	**5.6**
**37**	**127**	**QKDKLEKEVQEKEYNNDGL**	**5.4**
**142**	**156**	**QKETLEREVQNTQYNNETL**	**5.4**
**5**	**121**	**QKETLEREVQNTQYNNETL**	**5.4**
**46**	**183**	**QKENLEKEVAEATYKNETL**	**5.2**
**54**	**115**	**QRQNLEKEVAETKYKNETL**	**5**
**M2_enn127**	**132**	**KLEAINKELNENYYKLQDG**	**5**
**95**	**253**	**RLNAQERMYEAFLYQAKDI**	**4.7**
**14**	**134**	**QKERLEKKVQETEYNNGEL**	**4.7**
**74**	**141**	**QKETLERQVQEKEHNNEAL**	**4.6**
**14.4**	**162**	**QKEKLEKQVQEKEHNNEAL**	**4.6**
**19**	**163**	**QKETLERQVQEAQHNNNEL**	**4.5**
**49**	**88**	**ELEERQKNLEKLEHQSQVA**	**4.4**
**151**	**81**	**ELEERQKNLEKLEHQFQVA**	**4.4**
**47**	**150**	**QRENLEKEVAEAKHNNETL**	**4.4**
**207**	**109**	**QKENLEKEVAEAKHKNETL**	**4.4**
**18**	**108**	**QKENLEKEVAEAKHKNETL**	**4.4**
**55**	**270**	**EIQEKEAEKDRQQHMYEAF**	**4.3**
**222**	**262**	**EIQEKEAEKDRQQHMYEAF**	**4.3**
**164**	**88**	**EQQERQKKLEQLEHKYQVE**	**4.2**
**37**	**134**	**EVQEKEYNNDGLRHKNDDL**	**3.9**
**170**	**91**	**EQKERQKKLEQLEHKYQVE**	**3.9**
**M22_enn342**	**132**	**EQKERQKKLEQLEHKYQVE**	**3.9**
**M48_enn340**	**132**	**EQKERQKKLEQLEHKYQVE**	**3.9**
**M75_enn334**	**132**	**EQKERQKKLEQLEHKYQVE**	**3.9**
**M75_enn335**	**132**	**EQKERQKKLEQLEHKYQVE**	**3.9**
**M81_enn319**	**129**	**EQKERQKKLEQLEHKYQVE**	**3.9**
**M81_enn320**	**129**	**EQKERQKKLEQLEHKYQVE**	**3.9**
79	80	DYSQIEEKLEQFGHDYDKL	3.6
87	81	DYSEIEGKLEQFWHDYDKL	3.6
105	59	RADKLETENHGLKFQNEKL	3.4
105	143	QRVRLEKQVQEKEHNNKTL	3.4
207	60	RFEASDLENHKLKFDNDKL	3.4
79	108	QRVKLEKQVQEKEHNNKTL	3.4
218	122	QRVKLEKQVQEKEHNNKTL	3.4
100	59	KADKYEVRNHELEHNNEKL	3.4
209	80	DYSQIQEELEQFGHDYDKL	3.3
31	230	ENAKKDFELAALGHQLADK	3.25
229	224	ENAKKDFELAALGHQLADK	3.25
12	222	ENAKKDFELAALGHQLADK	3.25
228	208	ENAKKDFELAALGHQLADK	3.25
39	207	ENAKKDFELAALGHQLADK	3.25
193	200	ENAKKDFELAALGHQLADK	3.25
M11_enn344	132	EHKERQEKLEQLEHKYQVE	3.2
M63_enn346	132	EQKERQEKLEQLEHKYQVE	3.2
M63_enn327	129	EQKERQEKLEQLEHKYQVE	3.2
M63_enn324	129	EQKERQEKLEQLEHKYQVE	3.2
M63_enn325	129	EQKERQEKLEQLEHKYQVE	3.2
M85_enn329	129	EQKERQEKLEQLEHKYQVE	3.2
M85_enn330	129	EQKERQEKLEQLEHKYQVE	3.2

MB5_mrp70	61	EEVIANMSLDKLQHTLAGS	3.1
MB5_mrp71	61	EEVIANMSLDKLQHTLAGS	3.1
MB5_mrp72.0	61	EEVIANMSLDKLQHTLAGS	3.1
MB5_mrp79	61	EEVITNMSLEELQHTLAGS	3.1
39	57	EYHRLDTENHTLKHDKEKL	2.9
14	57	RAQDLEAKKHALEHQNTKL	2.9
14	57	RAQDLEAKNHGLEHQNTKL	2.9
63	44	EAQNNNSGKLTLEHKYNAL	2.8
MA2_mrp160	185	EAETLENLLGSAKHELTEL	2.7
MA2_mrp161	185	EAETLENLLGSAKHELTEL	2.7
26	122	NNKTLQTQNEDLTHENGQL	2.6
36	104	ELTEQNKELKAEEHRLITE	2.6
57	213	KLGQLNIDNIDLKHELEQE	2.6
57	185	ENQDLEEKLKDKEFYLGET	2.5
MB5_mrp55	56	REKALEEVIAKMPFEELQH	2.5
MB5_mrp56	56	REKALEEVIAKMPFEELQH	2.5
MB5_mrp57	56	REKALEEVIAKMPFEELQH	2.5
MB5_mrp58	56	REKALEEVIAKMPFEELQH	2.5
MB5_mrp62	56	REKALEEVIAKMPFEELQH	2.5
MA1_mrp282	189	EAATLENLVGSAKHELTDL	2.45
MA1_mrp284	189	EAATLENLVGSAKHELTDL	2.45
MA1_mrp287	189	EAATLENLLGSAKHELTEL	2.45
MA2_mrp115.0	185	EAATLENLLGSAKHELTDL	2.45
MA2_mrp117	185	EAATLENLLGSAKHELTDL	2.45
MA2_mrp118	185	EAATLENLLGSAKHELTDL	2.45
MA2_mrp119	185	EAATLENLLGSAKHELTDL	2.45
MA2_mrp136	185	EAATLENLLGSAKHELTDL	2.45
MA2_mrp139	185	EAATLENLLGSAKHELTDL	2.45
MA2_mrp140	185	EAATLENLLGSAKHELTDL	2.45
MA2_mrp141	185	EAATLENLLGSAKHELTGL	2.45
MA2_mrp143	185	EAATLENLLGSAKHELTGL	2.45
MA2_mrp144	185	EAATLENLLGSAKHELTGL	2.45
MA2_mrp145	185	EAATLENLLGSAKHELTGL	2.45
MA2_mrp155	185	EAATLENLLGSAKHELTEL	2.45
MA2_mrp156	185	EAATLENLLGSAKHELTEL	2.45
MA2_mrp159	185	EAATLENLLGSAKHELTEL	2.45
MA2_mrp167	185	EAATLENLLGSAKHELTDL	2.45
MA2_mrp168	185	EAATLENLLGSAKHELTDL	2.45
MA2_mrp171	185	EAATLENLLGSAKHELTDL	2.45
MA2_mrp172	185	EAATLENLLGSAKHELTEL	2.45
MA2_mrp174	185	EAATLENLLGSAKHELTDL	2.45
MA3_mrp293	220	EAATLENLLGSAKHELTDL	2.45
MA3_mrp295	220	EAATLENLLGSAKHELTDL	2.45
MA3_mrp296	220	EAATLENLLGSAKHELTDL	2.45
MA3_mrp297	220	EAATLENLLGSAKHELTEL	2.45
MA3_mrp298	220	EAATLENLLGSAKHELTEL	2.45
MB4_mrp6	226	EAATLENLLGSAKHELTEL	2.45
MB4_mrp7	226	EAATLENLLGSAKHELTEL	2.45
MB4_mrp8.0	226	EAATLENLLGSAKHELTEL	2.45
MB4_mrp16	226	EAATLENLLGSAKHELTDL	2.45
MB4_mrp19	226	EAATLENLLGSAKHELTDL	2.45
MB4_mrp20.0	226	EAATLENLLGSAKHELTDL	2.45
MB4_mrp22	226	EAATLENLLGSAKHELTDL	2.45
MB4_mrp23	226	EAATLENLLGSAKHELTDL	2.45
MB4_mrp25	226	EAATLENLLGSAKHELTDL	2.45

MB4_mrp26	226	EAATLENLLGSAKHELTDL	2.45
MB4_mrp27	226	EAATLENLLGSAKHELTDL	2.45
MB4_mrp30	226	EAATLENLLGSAKHELTDL	2.45
MB4_mrp31.1	226	EAATLENLLGSAKHELTDL	2.45
MB4_mrp34	226	EAATLENLLGSAKHELTDL	2.45
MB4_mrp36	226	EAATLENLLGSAKHELTDL	2.45
MB4_mrp37.1	226	EAATLENLLGSAKHELTDL	2.45
MB4_mrp39	226	EAATLENLLGSAKHELTDL	2.45
MB4_mrp40	226	EAATLENLLGSAKHELTEL	2.45
MB4_mrp41	226	EAATLENLLGSAKHELTDL	2.45
MB4_mrp42	226	EAATLENLLGSAKHELTDL	2.45
MB4_mrp43.0	225	EAATLENLLGSAKHELTEL	2.45
MB4_mrp45.0	225	EAATLENLLGSAKHELTEL	2.45
MB4_mrp46	225	EAATLENLLGSAKHELTEL	2.45
MB4_mrp48	225	EAATLENLLGSAKHELTEL	2.45
MB4_mrp49	225	EAATLENLLGSAKHELTEL	2.45
MB4_mrp50	225	EAATLENLLGSAKHELTDL	2.45
MB4_mrp53	225	EAATLENLLGSAKHELTDL	2.45
MB5_mrp88	216	EAATLENLLGSAKHELTEL	2.45
MB5_mrp89.1	216	EAATLENLLGSAKHELTEL	2.45
MB5_mrp91	216	EAATLENLLGSAKHELTEL	2.45
MB5_mrp93	216	EAATLENLLGSAKHELTDL	2.45
MB5_mrp94	216	EAATLENLLGSAKHELTDL	2.45
MB5_mrp96	216	EAATLENLLGSAKHELTDL	2.45
MB5_mrp98	216	EAATLENLLGSAKHELTEL	2.45
MB5_mrp99	216	EAATLENLLGSAKHELTEL	2.45
MB5_mrp100	216	EAATLENLLGSAKHELTEL	2.45
MB5_mrp101	216	EAATLENLLGSAKHELTEL	2.45
MB5_mrp102	216	EAATLENLLGSAKHELTEL	2.45
MB5_mrp103	216	EAATLENLLGSAKHELTEL	2.45
MB5_mrp55	216	EAATLENLLGSAKHELTEL	2.45
MB5_mrp56	216	EAATLENLLGSAKHELTEL	2.45
MB5_mrp57	216	EAATLENLLGSAKHELTEL	2.45
MB5_mrp58	216	EAATLENLLGSAKHELTDL	2.45
MB5_mrp59	216	EAATLENLLGSAKHELTEL	2.45
MB5_mrp61	216	EAATLENLLGSAKHELTDL	2.45
MB5_mrp63	216	EAATLENLLGSAKHELTDL	2.45
MB5_mrp66	216	EAATLENLLGSAKHELTDL	2.45
MB5_mrp68	216	EAATLENLLGSAKHELTEL	2.45
MB5_mrp69	216	EAATLENLLGSAKHELTEL	2.45
MB5_mrp70	216	EVATLENLLGSAKHELTEL	2.45
MB5_mrp71	216	EAATLENLLGSAKHELTEL	2.45
MB5_mrp72.0	216	EAATLENLLGSAKHELTEL	2.45
MB5_mrp75	216	EAATLENLLGSAKHELTDL	2.45
MB5_mrp76	216	EAATLENLLGSAKHELTDL	2.45
MB5_mrp77	216	EAATLENLLGSAKHELTDL	2.45
MB5_mrp79	216	EAATLENLLGSAKHELTEL	2.45
MB5_mrp80	216	EAATLENLLGSAKHELTEL	2.45
MB5_mrp82	216	EAATLENLLGSAKHELTDL	2.45
MB5_mrp86.0	216	EAATLENLLGSAKHELTEL	2.45
93	60	RLDEQNHKLVNDNHKLVND	2.4

Table S3. M6 FH-binding pattern in M, Enn and Mrp proteins.

Red horizontal line indicates cut-off.

M	Position	Sequence	Score
**6**	**123**	**NKELKAEENRLTTENKGLTKKLSEAEEEAANKERE**	**6.4**
**36**	**109**	**NKELKAEEHRLITENRGLTKKLSEAEEESVNKERE**	**6.4**
**213**	**95**	**NEDLTREYRRLTQDNRGLTQKNEDLTQKNHRLTQE**	**6.4**
**71**	**95**	**NEDLTQEKQRLTSENRGLTKENEDLTQKNHRLTQD**	**6.4**
**32**	**67**	**NHQLTQENEKLTQENEKLTKDKEELTQENEKLTKD**	**6.1**
**183**	**56**	**YSQLHDDYDKLQEQNGEYLKKIGELEERQKNLEKL**	**5.8**
**238**	**57**	**ANNTTVQNIRLRNENKNLKAKNEDLEARLENAMNV**	**5.8**
**17**	**88**	**NEELGQEKEKLGQENEELKQEKEKLKTQAAELEET**	**5.7**
**17**	**123**	**NREYGAEKDRLVLENRDLENKNRDLENKNRDLEGG**	**5.7**
**26**	**144**	**NKDYEAENGRLESDNRRLEGKNKDLEGKNKDLEGK**	**5.7**
**115**	**116**	**NEDLTREYDRLTQENRGLTQDKDELSKQKETLGLA**	**5.7**
**30**	**165**	**KEDLTREYRRLTQDNRGLTKDREDLTQKNHELSGQ**	**5.7**
**197**	**193**	**KEDLTREYRRLTQDNRGLTKDREDLTQKNHELSGQ**	**5.7**
M18_enn300	96	KWNLNDEYNKLLDENEKLKEEIGGYLDKQEQLEQL	5.4
M64_enn306	96	KWNLNDEYNKLLDENEKLKEEIGGYLDKQEQLEQL	5.4
M80_enn310	96	KWNLNDEYNKLLDENEKLKEEIGGYLDKQEQLEQL	5.4
M80_enn311	96	KWNLNDEYNKLLDENEKLKEEIGGYLDKQEQLEQL	5.4
M98_enn314	96	QWNLTEEYNKLHEENERLKEEIGGYLDKQDQLEQL	5.4
M101_enn300	96	KWNLNDEYNKLLDENEKLKEEIGGYLDKQEQLEQL	5.4
M123_enn300.1	96	KWNLNDEYNKLLDENEKLKEEIGGYLDKQEQLEQL	5.4
158	67	RDNLLGENGKLWDENETLREKQEELEKENEKLDSQ	5.4
205	55	KWNLNDEYNKLLDENEKLKEEIGGYLDKQEQLEQL	5.4
117	59	YNELSGEYNKLLDQNGNLLDENEILKEKLDKDQEE	5.4
100	67	NHELEHNNEKLKTENSDLKTENSKLTSEKEELTQE	5.4
M54_enn260	87	QFDWEKEYKKLDEDNAKLVEVVEATSLENEKLKSE	5.2
M71_enn263	87	QFDWEKEYKKLDGDNAKLVEVVEATSLENEKLKSE	5.2
M71_enn262	87	QFDWEKEYKKLDGDNAKLVEVVEATSLENEKLKSE	5.2
MA1_mrp247	113	LNNKNEQIAKLTNENAQLKEAVEGYVQTIQNASRE	5.1
MA1_mrp248	113	LNNKNEQIAKLTNENAQLKEAVEGYVQTIQNASRE	5.1
MA1_mrp278	113	LNNKNEQIAKLTNENAQLKEAVEGYVQTIQNASRE	5.1
MA1_mrp279	113	LNNKNEQIAKLTNENAQLKEAVEGYVQTIQNASRE	5.1
166	48	QVDWEKEYKKLDEDNAKLVEVVETTSLENEKLKSE	5
47	67	IHQLKDDKEKLQSQNENLQSQNENLQSQNENLQSQ	4.9
79	88	LEQFGHDYDKLEKENKEYASQLGKNQEEREKLELE	4.7
209	88	LEQFGHDYDKLEKENKEYASQLGKNQEEREKLELG	4.7
87	89	LEQFWHDYDKLEKENKEYASQLGKNQEEREKLELE	4.7
103	92	LEQFGRDYDKLEKENKEYASQLGKNQEEREKLELE	4.7
157	51	REQLRQEHDRLEAENSKLLNQTEKLQKKITDLTTE	4.7
147	51	KEQLRQEHDRLEIENHKLLNETEKLQKKITDLNTK	4.7
